# Femoral artery pseudoaneurysm in Behçet's syndrome: long-term outcomes and associated factors

**DOI:** 10.3389/fcvm.2026.1942219

**Published:** 2026-10-02

**Authors:** Yanfei Yang, Yuexin Chen

**Affiliations:** Department of Vascular Surgery, Peking Union Medical College Hospital, Chinese Academy of Medical Sciences & Peking Union Medical College, Beijing, China

**Keywords:** Behçet's syndrome, femoral artery pseudoaneurysm, immunosuppressive adherence, individual patient data meta-analysis, inflammatory indices

## Abstract

**Background:**

Behçet's syndrome with femoral artery pseudoaneurysm (BS–FAP) is exceedingly rare, and its long-term postoperative recurrence profile remains undefined. We aimed to ascertain event-free survival (EFS) and examine associations of immunosuppressive adherence and five inflammatory biomarkers (*N*LR, PLR, SII, SIRI, IBI) with relapse. External validation was performed via systematic review and individual patient data (IPD) meta-analysis.

**Methods:**

This retrospective study included 13 BS–FAP patients who underwent femoral artery surgery at Peking Union Medical College Hospital (2002–2022). The primary endpoint was EFS, a composite of same-site recurrence, *de novo* aneurysm, re-intervention, death, or amputation. Five inflammatory indices were derived from preoperative blood counts. EFS was estimated using Kaplan–Meier with log-rank comparisons. A systematic literature search was conducted for descriptive IPD meta-analysis.

**Results:**

The cohort (median age 34 years, 85% male) underwent endovascular (*n* = 6), open (*n* = 6), or hybrid (*n* = 1) repair. Median follow-up was 60 months (all ≥36 months). Five-year EFS was 69.2% in primary analysis and 76.9% in sensitivity analysis. Endovascular and open approaches showed comparable EFS (*p* = 0.992). Elevated NLR, PLR, SIRI, and IBI were associated with lower 5-year EFS (50.0% vs. 85.7%), with SIRI showing the strongest trend (*p* = 0.131); SII (*p* = 0.787) and ESR (*p* = 0.994) showed no trend. Three patients (23.1%) developed *de novo* aneurysms beyond 5 years, all after immunosuppressive discontinuation. In a supplementary four-level adherence analysis, no patient was fully adherent perioperatively; preoperative-only use (*n* = 2) showed the highest disease-specific 5-year EFS (100%). The cohort showed mucocutaneous predominance (oral ulcers 92.3%) and venous involvement in 38.5%. IPD meta-analysis (40 individual cases + 12 series) confirmed a temporal mortality decline from 26%–40% (early open) to 0%–5.8% (contemporary endovascular). Published case reports (median follow-up 6 months) reported 11% recurrence, vs. 31% in our 60-month cohort, confirming systematic underestimation.

**Conclusion:**

The recurrence burden after BS–FAP surgery is systematically underestimated in short-follow-up literature; surveillance should extend beyond 5 years. Immunosuppressive adherence is a modifiable target for recurrence control. SIRI emerges as the most promising biomarker for risk stratification. Endovascular and open repair offer comparable long-term EFS. This exploratory study supports a comprehensive pathway of “surgical intervention—uninterrupted immunosuppression—extended surveillance,” with adherence incorporated into quality metrics.

## 1 Introduction

Behçet's syndrome (BS) is a rare systemic vasculitis of unknown etiology, characterized by recurrent oral ulcers, genital ulcers, ocular lesions, and cutaneous manifestations (1). BS is highly prevalent along the ancient Silk Road, with an estimated prevalence of 80–370 per 100,000 in Turkey, approximately 14 per 100,000 in China, and only 0.3–7.5 per 100,000 in Europe and the United States (1, 2). Although BS itself is not uncommon in endemic regions, vascular involvement (vasculo-BS) shows considerable variation in reported frequency-approximately 1%–7% in some series but as high as 25%–40% in cohorts from highly endemic regions-with venous involvement (thrombosis/phlebitis) consistently more common than arterial disease (3–6). Vasculo-BS represents a severe phenotype of the syndrome, and aneurysm and pseudoaneurysm are the leading causes of death, with early open surgical series reporting mortality rates as high as 26%–40% (7, 8).

Among arterial involvement in vasculo-BS, the aorta and pulmonary artery are the most frequently affected sites, whereas femoral artery pseudoaneurysms are relatively common among peripheral arterial pseudoaneurysms but remain extremely rare in absolute case numbers. In the global literature, BS complicated by femoral artery pseudoaneurysm (FAP) has been reported almost exclusively as single case reports or small series of fewer than 5 cases, with no intermediate- or long-term (≥3 years) follow-up data available (5, 6, 9, 10). In other words, BS–FAP represents a “rare entity within a rare disease”—BS itself is a rare disease, vasculo-BS is a rare subtype of BS, and femoral artery pseudoaneurysm is an uncommon site among peripheral vascular involvement in vasculo-BS. This double or even triple rarity precludes any single center from accumulating a large cohort, leaving high-quality evidence in long-standing deficit.

Consequently, two key clinical questions remain unresolved. First, what is the true long-term postoperative recurrence rate of BS–FAP? Second, what roles do perioperative and maintenance immunosuppressive therapy, as well as objective inflammatory indices, play in recurrence control and risk stratification? The pathogenesis of BS arterial aneurysms is characterized by inflammatory destruction of the vessel wall, distinct from atherosclerotic aneurysms, with high rates of postoperative anastomotic pseudoaneurysm and graft thrombosis, suggesting that inflammatory activity is the core driver of recurrence (7, 11). In recent years, a panel of peripheral blood-derived inflammatory indices—including neutrophil-to-lymphocyte ratio (NLR), platelet-to-lymphocyte ratio (PLR), systemic immune-inflammation index (SII), systemic inflammation response index (SIRI), and inflammation-based index (IBI)—have been validated as surrogates of systemic inflammation–immune imbalance and have been shown to predict adverse outcomes in various vasculitides, atherosclerosis, and malignancies (12, 13). However, the value of these indices in predicting postoperative recurrence of BS–FAP has not been systematically investigated.

Our center previously reported a study on the timing of surgical intervention for pseudoaneurysms in 96 patients with vasculo-BS (1), confirming that surgery significantly reduces aneurysm-related mortality and proposing a surgical threshold of >4 cm for aortic aneurysms and >2.5 cm for peripheral aneurysms. Nevertheless, the long-term event-free survival, recurrence patterns, and associations with immunosuppressive adherence and inflammatory indices in the femoral artery subgroup have not been analyzed in depth. Moreover, that study had a data cutoff in December 2021 and did not capture subsequent late-onset events in this cohort.

The rationale for these hypotheses rests on three lines of evidence. First, BS vasculitis is chronic and relapsing: histopathological and angiographic studies show that arterial lesions evolve over years, with new aneurysms often appearing long after the index lesion, so that short follow-up systematically underestimates the true recurrence burden (3, 4, 11). Second, non-adherence to immunosuppressive therapy is a recognized driver of vasculo-BS relapse: vessel-wall inflammation is T-cell-, neutrophil- and monocyte-mediated, and glucocorticoids combined with disease-modifying antirheumatic drugs (DMARDs) suppress this inflammatory microenvironment, so treatment interruption permits inflammatory rebound and late pseudoaneurysm formation (3, 4, 11). Third, peripheral-blood inflammatory indices-including NLR, PLR, SII, SIRI and IBI-have been validated as surrogates of systemic inflammation-immune imbalance and predict adverse outcomes in vasculitis, atherosclerosis and malignancy (12, 13) providing a biologically plausible basis for their predictive value in BS-FAP recurrence.

On this basis, the present study leveraged an extended follow-up cohort of 13 patients with BS–FAP, complemented by a literature-based IPD meta-analysis, aiming to: (1) determine the long-term EFS after BS–FAP surgery; (2) evaluate the associations of immunosuppressive adherence and multiple inflammatory indices with recurrence; and (3) externally contextualize our cohort against the global literature. We hypothesize that extended follow-up will uncover a recurrence burden underestimated by short-term studies, and that non-adherence to immunosuppressive therapy and elevated inflammatory indices—particularly SIRI—are associated with postoperative recurrence or *de novo* aneurysm formation. Given the rarity of BS–FAP, this study is positioned as exploratory and hypothesis-generating.

## 2 Methods and data

### 2.1 Study design and population

This study is reported in accordance with the Strengthening the Reporting of Observational Studies in Epidemiology (STROBE) statement. The study design was a single-center retrospective cohort study, approved by the Institutional Review Board of Peking Union Medical College Hospital, with a waiver of informed consent. Inclusion criteria were: (1) fulfillment of the International Criteria for Behçet's Disease (ICBD) diagnostic criteria; (2) underwent surgical treatment for femoral artery pseudoaneurysm as the primary intervention site at Peking Union Medical College Hospital between January 2002 and May 2022; and (3) complete baseline complete blood count and C-reactive protein (CRP) data available for calculation of derived inflammatory indices. Exclusion criteria were: (1) missing baseline key variables (complete blood count, CRP) precluding calculation of inflammatory indices; (2) lost to follow-up postoperatively with inability to ascertain endpoint events through outpatient visits or telephone contact; and (3) underwent percutaneous drainage or conservative medical therapy without surgical intervention.

Screening flow (Figure 1): Among the vascular-BS database at our center, 43 patients with BS and lower extremity aneurysms were identified → 29 underwent femoral artery surgery → 18 had femoral artery pseudoaneurysm as the primary treatment site → 5 were excluded due to missing key data → 13 were finally enrolled.

**Figure 1:**
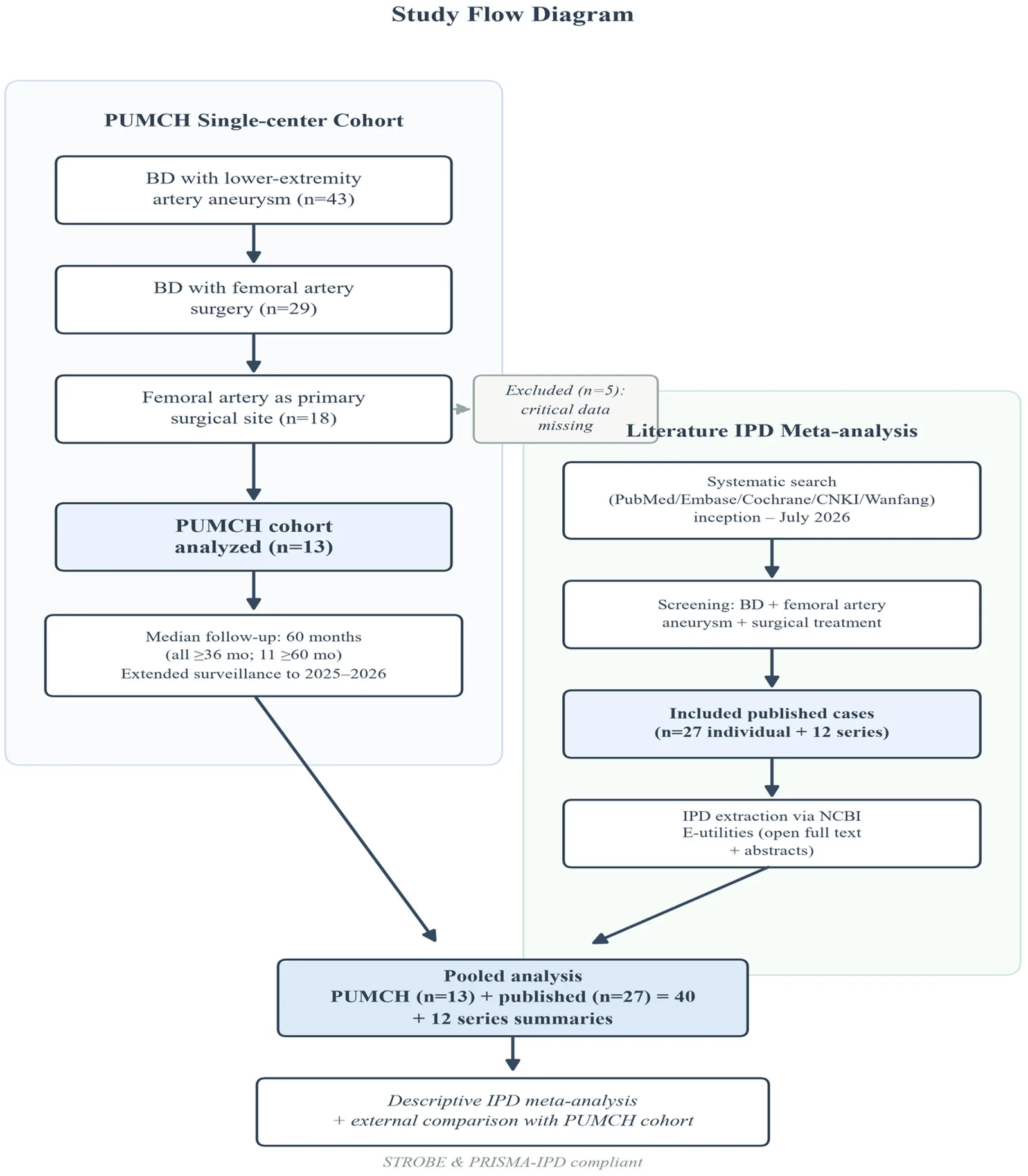
Study flowchart. From 43 patients with BS and lower extremity aneurysms, 13 were ultimately enrolled after sequential screening for femoral artery surgery (*n* = 29), femoral artery as the primary treatment site (*n* = 18), and exclusion of 5 cases with missing key data. The IPD meta-analysis pooled 27 published individual cases with the 13 cases from our cohort (overall 40 cases), alongside 12 case series summaries.

Position statement: This study represents a focused subgroup analysis of the femoral artery cohort derived from our center's previously published vasculo-BS series (1). However, the research questions are fundamentally distinct from the parent study: the parent study focused on surgical timing and overall survival, whereas the present study focuses on long-term postoperative event-free survival, immunosuppressive adherence–recurrence associations, predictive value of inflammatory indices, and IPD-based external comparison. Moreover, follow-up has been extended in the present study (with some patients having monitoring prolonged to 2025–2026 triggered by late events). The two studies are independent in their research questions, methodologies, and conclusions, with distinct emphases.

### 2.2 Variables and outcomes

Baseline data were extracted from inpatient medical records and outpatient follow-up systems, including demographic characteristics (age, sex), BS clinical phenotypes (oral ulcers, genital ulcers, ocular lesions, skin lesions, positive pathergy test, venous thrombosis/phlebitis), BS disease duration (from initial BS symptom onset to FAP surgery date), and cardiovascular risk factors (hypertension, diabetes, smoking history). Surgical variables included procedure type, categorized as endovascular repair (stent-graft implantation), open repair (synthetic bypass grafting or patch plasty), and hybrid procedures (combined endovascular and open techniques). Aneurysm site (common femoral artery, superficial femoral artery, deep femoral artery, or bifurcation), maximum aneurysm diameter (measured on preoperative CTA), and technical success (intraoperative confirmation of pseudoaneurysm exclusion/resection with no endoleak or residual flow on immediate angiography or intraoperative ultrasound) were recorded.

For each patient, the full spectrum of BS manifestations was recorded, including mucocutaneous involvement (oral and genital ulcers, skin lesions), ocular lesions, positive pathergy test, venous involvement (deep-vein thrombosis or superficial phlebitis), gastrointestinal, neurologic and articular involvement, and concomitant aneurysms at other arterial sites (including pulmonary arterial aneurysm). These phenotypic data were collected to contextualize the inflammatory indices and recurrence patterns.

Laboratory indices were measured from morning fasting peripheral venous blood samples collected within 1 week preoperatively, tested uniformly by the Department of Clinical Laboratory at Peking Union Medical College Hospital. Recorded parameters included CRP (mg/L), ESR (mm/h), D-dimer (mg/L), white blood cell count (WBC, ×10^9^/L), absolute neutrophil count (×10^9^/L), absolute monocyte count (×10^9^/L), and platelet count (PLT, ×10^9^/L).

Derived inflammatory indices were calculated from preoperative complete blood counts, including five indices.

- NLR = neutrophil count/lymphocyte count
- PLR = platelet count/lymphocyte count
- SII = neutrophil count × platelet count/lymphocyte count
- SIRI = neutrophil count × monocyte count/lymphocyte count
- IBI = CRP (mg/dL) × NLR, with CRP converted from mg/L to mg/dL by division by 10

Reference ranges of the laboratory indices used in this study (Department of Clinical Laboratory, Peking Union Medical College Hospital) were: CRP 0–8 mg/L; ESR 0–15 mm/h in men and 0–20 mm/h in women; WBC 3.5–9.5 × 10^9^/L; neutrophil count 1.8–6.3 × 10^9^/L; lymphocyte count 1.1–3.2 × 10^9^/L; monocyte count 0.1–0.6 × 10^9^/L; platelet count 125–350 × 10^9^/L; D-dimer 0–0.5 mg/L. Reported reference values for the derived indices in healthy adults are approximately NLR 1.0–3.0, PLR 100–200, SII 300–700, SIRI 0.5–2.0, and IBI 0–10 (12, 13).

Assessment of Immunosuppressive Therapy:

Preoperative status: Record whether immunosuppressive therapy [glucocorticoids ± disease-modifying antirheumatic drugs (DMARDs)] had been initiated before surgery, and its duration.

Postoperative status: Record immunosuppressive maintenance status from surgery to the last follow-up.

Operational definition of “adherent”: Continuous use of glucocorticoids (prednisone ≥5 mg/day or equivalent) with or without DMARDs (including cyclophosphamide, azathioprine, cyclosporine, mycophenolate mofetil, tocilizumab, infliximab, etc.) from at least 2 weeks before surgery to the last follow-up, without self-discontinuation or unauthorized dose reduction exceeding 50% for more than 2 weeks. Any patient not meeting the above criteria (including never used, used preoperatively but discontinued postoperatively, unauthorized dose reduction beyond threshold, or self-discontinuation) was classified as “non-adherent.” Preoperative and postoperative statuses were recorded separately.

To address the limitation of a dichotomous adherence classification and to capture adherence as a continuum, a supplementary four-level adherence variable was constructed by combining preoperative and postoperative status: (A) fully adherent-regular immunosuppression both before and after surgery; (B) preoperative-only-regular preoperatively but not maintained postoperatively; (C) postoperative-only-not regular preoperatively but regular postoperatively; and (D) neither-regular in neither period. This four-level variable was used in a supplementary descriptive survival analysis.

Outcome Measures:

The primary endpoint was event-free survival (EFS), defined as the time from surgery to the first occurrence of any component of the composite endpoint. The composite endpoint comprised: ① same-site recurrence (pseudoaneurysm or anastomotic aneurysm at the original surgical site); ② *de novo* occurrence at a new site (new pseudoaneurysm in the femoral artery or other peripheral arteries at a different site); ③ re-intervention (secondary surgery or intervention due to the above events or technical complications); ④ all-cause death; and ⑤ amputation (at any level of the affected lower limb). The earliest event was taken as the endpoint.

Secondary endpoints included technical success rate, perioperative complications (within 30 days postoperatively), length of hospital stay, and recurrence pattern (same-site vs. *de novo*).

Definition of technical complications (prespecified for sensitivity analysis): Technical complications were defined as adverse events directly related to surgical procedure rather than disease (BS vasculitis) progression, including but not limited to: in-stent thrombosis, graft occlusion, puncture-site or anastomotic hematoma requiring secondary intervention, and graft infection. In the sensitivity analysis, re-interventions due to technical complications were excluded from the composite endpoint, retaining only disease-specific events (same-site recurrence/*de novo* occurrence/death/amputation), to estimate disease-specific EFS and avoid dilution of disease recurrence assessment by technical factors. In this study, graft or in-stent thrombosis, graft occlusion, and other mechanical/procedure-related events occurring within ≤3 months postoperatively were uniformly defined as technical complications, and were strictly distinguished from *in situ* recurrence occurring at ≥6 months postoperatively due to BS vasculitis activity.

### 2.3 IPD meta-analysis

Design positioning: The IPD meta-analysis in this study was positioned as a descriptive external control rather than a traditional two-stage or one-stage meta-analysis with pooled effect sizes. The rationale was that most published literature consisted of case reports with heterogeneous follow-up durations and inconsistent definitions of recurrence, precluding statistically meaningful effect-size pooling. This analysis aimed to contextualize the baseline characteristics, procedural distribution, and outcomes of our cohort against the global literature, allowing for temporal trend comparisons and follow-up duration contrasts, rather than validating the inflammation–recurrence hypothesis, which was tested exclusively in our cohort.

Search strategy: A systematic search was performed in PubMed, Embase, Cochrane Library, CNKI (China National Knowledge Infrastructure), and Wanfang databases, covering the period from inception to July 18, 2026. The search strategy (using PubMed as an example) was as follows:

text

(“Behçet disease” OR “Behcet disease” OR “Behçet’s syndrome” OR “Behcet’s syndrome” OR “BD” OR “vasculo-Behçet”) AND (“femoral artery” OR “femoral pseudoaneurysm” OR “femoral aneurysm” OR “SFA” OR “profunda femoris”) AND (“surgery” OR “repair” OR “endovascular” OR “stent” OR “bypass” OR “graft” OR “embolization” OR “intervention”)

The search term “Behçet's syndrome” was included because some authors and the EULAR task force use “syndrome” rather than “disease”.

Inclusion criteria: ① Diagnosis of BS (explicitly using ICBD/Japanese/International criteria) with femoral artery aneurysm (true or pseudoaneurysm, no restriction); ② Underwent surgical treatment (open/endovascular/hybrid); ③ At least 1 case or case series reported. Exclusion criteria: ① Non-femoral sites (aorta, iliac artery, popliteal artery only, etc.); ② Non-BS etiology (e.g., trauma, infection, atherosclerosis); ③ Duplicate publications or overlapping data (only the most complete or recent version was included).

Data extraction: Two investigators independently extracted data, with disagreements resolved by consensus. A standardized extraction form was designed, containing 31 data fields:

- Literature information: first author, year of publication, country, journal, document type (case report/small series)
- Patient baseline: age, sex, BS duration, aneurysm type (true/pseudo), maximum aneurysm diameter, site
- Surgical information: procedure type (open/endovascular/hybrid), urgency (emergency/elective)
- Immunosuppression information: preoperative and/or postoperative use of glucocorticoids and DMARDs (extracted whenever possible)
- Outcome information: follow-up duration (months), recurrence/*de novo* occurrence (time, site), re-intervention, death, amputation

Data source stratification: Individual patient data were preferentially extracted directly from the full text or tables. If a study reported only summary statistics (e.g., “mortality rate of 26% in the series”), these data were incorporated as series-level summary data for heatmap and forest plot comparisons, but were not included in individual-level descriptive statistics.

Data pooling strategy: The 13 cases from our cohort were combined with published individual cases into a “pooled individual data set (*n* = 40)” for descriptive baseline and outcome analyses. Summary rate values from 12 case series were used to generate temporal trend heatmaps and mortality forest plots. The two data types were labeled separately in figures and were not analyzed together.

### 2.4 Statistical analysis

All statistical analyses were performed using R software (version 4.3), with the survival, survminer, and meta packages. All statistical tests were two-sided, with a prespecified significance level of *α* = 0.05. However, given the small sample size and the exploratory nature of this study, *p*-values were used as auxiliary references and were not interpreted as rigid determinants of statistical significance.

Descriptive statistics: Continuous variables were presented as medians with interquartile ranges (IQR), and categorical variables as counts with percentages. The baseline characteristics table was descriptively presented without between-group hypothesis testing; differences in direction and magnitude between groups were described narratively, with selection bias explicitly identified.

Survival analysis: Event-free survival curves were estimated using the Kaplan–Meier method, with between-group comparisons performed via the log-rank test. Follow-up duration was estimated using the reverse Kaplan–Meier method (i.e., censoring and events interchanged), which avoids overestimation of median follow-up due to early loss to follow-up and provides a more accurate reflection of the actual observation time of the cohort. Patients lost to follow-up were censored at their last known follow-up time in Kaplan–Meier analyses; intraoperative death (none in this cohort) would have been counted as an event. Subgroup variables for the log-rank test included: procedure type (endovascular vs. open), preoperative immunosuppressive adherence, CRP median group, ESR median group, and median groups for each of the five inflammatory indices. The single hybrid case was excluded from procedure-type subgroup comparisons.

Sensitivity analysis: To evaluate the dilution effect of technical complications on EFS, a sensitivity analysis was performed by excluding re-interventions due to technical complications from the composite endpoint (see “Definition of technical complications”), and disease-specific EFS was re-estimated.

Multiple comparisons and adjustment strategy: This study involved log-rank tests for five inflammatory indices (five comparisons), raising a multiple comparisons issue. In theory, adjustment (e.g., Bonferroni correction) should be applied. However, for the following considerations, no formal adjustment was performed: ① The extremely small sample size (*n* = 13) and event count (<10) render multiplicity adjustment overly conservative, with a high risk of type II error in detecting genuine trends; ② This study is explicitly positioned as exploratory and hypothesis-generating rather than confirmatory; ③ The five inflammatory indices are collinear (all derived from the same complete blood count) and are not independent tests, making Bonferroni correction excessively stringent. Therefore, the direction of effect, absolute between-group differences (e.g., 5-year EFS 50.0% in high-value group vs. 85.7% in low-value group), and 95% confidence intervals were used as the primary basis for interpretation, with *p*-values serving only as a reference for the strength of trends rather than the sole criterion for “statistical significance.” This strategy aligns with methodological recommendations for rare disease research.

Exemption statement for regression modeling: Due to the event count (*n* = 4) being substantially lower than the conventional rule of thumb (at least 10–15 events per variable), multivariate Cox regression could not be performed. Forced fitting would result in model overfitting and unreliable parameter estimates. Consequently, no multivariable regression analysis was conducted in this study; all subgroup analyses were univariate stratified comparisons. This limitation is further addressed in the Discussion.

Supplementary four-level adherence analysis: The four-level adherence variable was analyzed with the Kaplan–Meier method and log-rank test across groups B, C and D (group A was not estimable as no patient met full-adherence criteria); given the extremely small cell counts, this analysis is descriptive only and no formal inferential claim is made.

IPD meta-analysis statistics: Pooled individual case data were described as medians (IQR) or rates (%); case series rates were cited directly from original reports, with heatmaps and bar forest plots constructed to display temporal trends. No meta-analytic pooling of rates was performed (given excessive heterogeneity, with *I*^2^ anticipated to exceed 90%).

Data availability statement: Due to ethical restrictions and patient privacy protection, the individual patient data from this study cannot be publicly shared in an open database. Reasonable requests may be directed to the corresponding author and will be considered upon institutional review board approval.

## 3 Results

### 3.1 Baseline characteristics and selection bias

Baseline characteristics of the 13 patients are summarized in Table 1. The median age was 34 years (IQR 29–48), with 11 male patients (85%). All aneurysms were pseudoaneurysms. The involved sites were: common femoral artery in 5, superficial femoral artery in 4, deep femoral artery in 2, and bifurcation in 2. The median maximum aneurysm diameter was 5.2 cm. Six patients underwent endovascular repair, six open surgery, and one a hybrid procedure. The median BS duration was 4 years (IQR 2–5); however, only 2 patients (15.4%) had received regular immunosuppressive therapy before BS-FAP surgery, while 5 (38.5%) had documented venous involvement and 3 (23.1%) had concomitant aneurysms at other arterial sites, indicating that in most patients the femoral pseudoaneurysm arose in the context of established but undertreated BS rather than as the inaugural manifestation.

**Table 1:** Baseline characteristics of the 13 patients with BS–FAP (descriptive).

Variable	Endovascular (*n* = 6)	Open (*n* = 6)	Hybrid (*n* = 1)
Age (years), median (IQR)	32.0 (30.0–35.5)	43.5 (31.5–48.8)	31
Male, *n* (%)	5 (83.3)	5 (83.3)	1 (100)
Maximum aneurysm diameter (cm)	5.7 (4.7–8.5)	5.2 (3.8–6.3)	10.9
Site (CFA/SFA/DFA/bifurcation)	3/2/0/1	2/1/1/2	1/0/0/0
Preoperative regular immunosuppression, *n* (%)	2 (33.3)	0 (0)	0 (0)
CRP (mg/L)	6.1 (1.0–30.1)	36.8 (22.1–127.4)	2.8
ESR (mm/h)	14.5 (6.2–25.0)	46.0 (23.5–56.5)	6
NLR	2.30 (1.83–3.05)	3.85 (2.45–6.20)	2.10
PLR	78.5 (55.2–105.3)	148.2 (82.5–225.6)	95.0
SII	420 (350–580)	780 (520–2,200)	480
SIRI	1.85 (1.20–2.30)	2.80 (1.90–3.50)	1.60
IBI	2.5 (0.5–8.2)	28.0 (15.0–52.0)	0.7
D-dimer (mg/L)	2.1 (1.5–2.5)	2.7 (1.5–4.0)	7.6
Pseudoaneurysm (ruptured)	6(100)	6(100)	1(100)

Data are presented as median (IQR) or *n* (%). Given the small sample size inherent to the rarity of BS–FAP, this table is presented descriptively without between-group hypothesis testing. The endovascular group was younger and had a lower inflammatory burden (CRP/ESR/SIRI, etc.) compared with the open group, suggesting a clinical selection bias—patients with milder inflammation were more likely to be selected for endovascular repair—which constitutes an important context for interpreting procedure-type comparisons. NLR, neutrophil-to-lymphocyte ratio; PLR, platelet-to-lymphocyte ratio; SII, systemic immune-inflammation index; SIRI, systemic inflammation response index; IBI, inflammation-based index (CRP mg/dL × NLR); CRP, C-reactive protein; ESR, erythrocyte sedimentation rate. All cases were pseudoaneurysms.

The preoperative inflammatory burden was generally elevated: median CRP was 21.06 mg/L (elevated in 9/13 patients), and median ESR was 22 mm/h (elevated in 7/13 patients). The derived inflammatory indices were as follows: median NLR 2.80, PLR 98.01, SII 547.56, SIRI 2.46, and IBI 13.61. Of note, the endovascular group was younger (median 32 vs. 43 years) and had a lower inflammatory burden (median CRP 6.1 vs. 36.8 mg/L, ESR 14.5 vs. 46.0 mm/h, SIRI 1.85 vs. 2.80) compared with the open group, suggesting a clinical preference for endovascular repair in patients with milder inflammation and younger age—a selection bias that constitutes an important context for interpreting subsequent procedure-type comparisons.

The full phenotypic spectrum of the 13 patients is summarized in Supplementary Table S1. Mucocutaneous involvement predominated: oral ulcers in 12 (92.3%) and genital ulcers in 7 (53.8%). Ocular lesions were present in 5 (38.5%) and skin lesions in 4 (30.8%); pathergy test was positive in 3 (23.1%). Venous involvement (deep-vein thrombosis or superficial phlebitis) was documented in 5 (38.5%), and gastrointestinal involvement in 3 (23.1%); neither neurologic nor articular involvement was recorded. Three patients (23.1%) had concomitant aneurysms at other arterial sites (popliteal, abdominal aortic, and other peripheral arteries); no pulmonary arterial aneurysm was identified.

### 3.2 Surgical and perioperative outcomes

All 13 patients underwent successful procedures, with a technical success rate of 100%. Perioperative complications occurred in 2 cases: case 2 developed graft thrombosis at 1 month postoperatively (technical complication, treated with thrombectomy), and case 3 had delayed wound healing; both resolved with management. The median postoperative hospital stay was 13 days. One re-intervention occurred within 30 days (case 2). There were no 30-day deaths or perioperative amputations.

### 3.3 Long-Term event-free survival

The median follow-up duration was 60 months (range 36–60; all patients >=36 months, 11 ≥ 60 months). In the primary analysis using the first-event principle, 4 patients experienced events within 5 years: case 2 developed a technical complication at 1 month (graft thrombosis with thrombectomy; open repair, non-adherent); case 10 had a technical complication at 3 months (in-stent thrombosis with catheter-directed thrombolysis; endovascular repair, adherent preoperatively but discontinued postoperatively); case 13 developed a *de novo* aneurysm at a new site at 12 months (endovascular repair, non-adherent); and case 6 had *in situ* recurrence at 24 months (open repair, non-adherent). The early thrombotic events in cases 2 and 10 were classified as technical complications (≤3 months postoperatively). The 24-month *in situ* recurrence in case 2 occurred after the initial event and was not counted in the primary analysis but was included as a disease-specific event in the sensitivity analysis. The primary analysis yielded a 5-year EFS of 69.2% (1-year 76.9%; 2-year 69.2%; 3-year 69.2%) (Figure 2A). In the sensitivity analysis excluding technical complications (case 2 at 1 month and case 10 at 3 months), the 24-month *in situ* recurrence in case 2 was re-included, giving a disease-specific 5-year EFS of 76.9% (1-year 92.3%) (Figure 2B).

**Figure 2:**
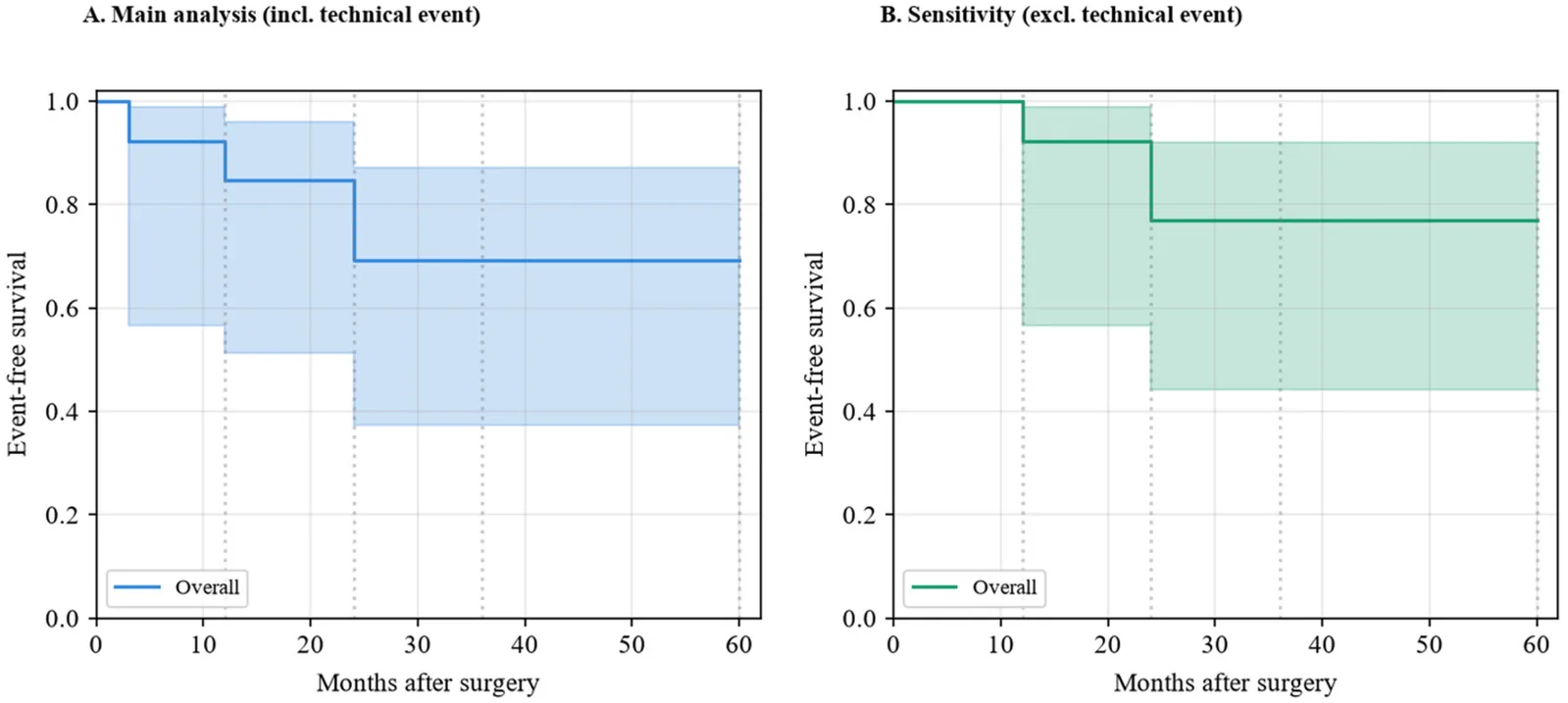
Kaplan–meier curves for event-free survival (EFS). **(A)** Primary analysis (including technical complications), 5-year EFS 69.2%. **(B)** Sensitivity analysis (excluding technical complications), 5-year EFS 76.9%. Shaded areas represent 95% confidence intervals. Dashed vertical lines indicate time points at 12, 24, 36, and 60 months.

### 3.4 Drivers of recurrence

The 5-year EFS was 66.7% for both endovascular and open approaches (log-rank *p* = 0.992), with no significant difference. The endovascular group had lower baseline inflammation and was younger (see 3.1), which may confound this comparison.

Given that only 2 patients were in the adherent group, the primary analysis (5-year EFS 50.0% adherent vs. 72.7% non-adherent, *p* = 0.462) had very limited statistical power; the lower EFS in the adherent group was largely driven by the technical complication in case 10. In the sensitivity analysis excluding technical complications, the 5-year EFS in the adherent group rose to 100% vs. 72.7% in the non-adherent group (*p* = 0.431, Figures 3A,B).

**Figure 3:**
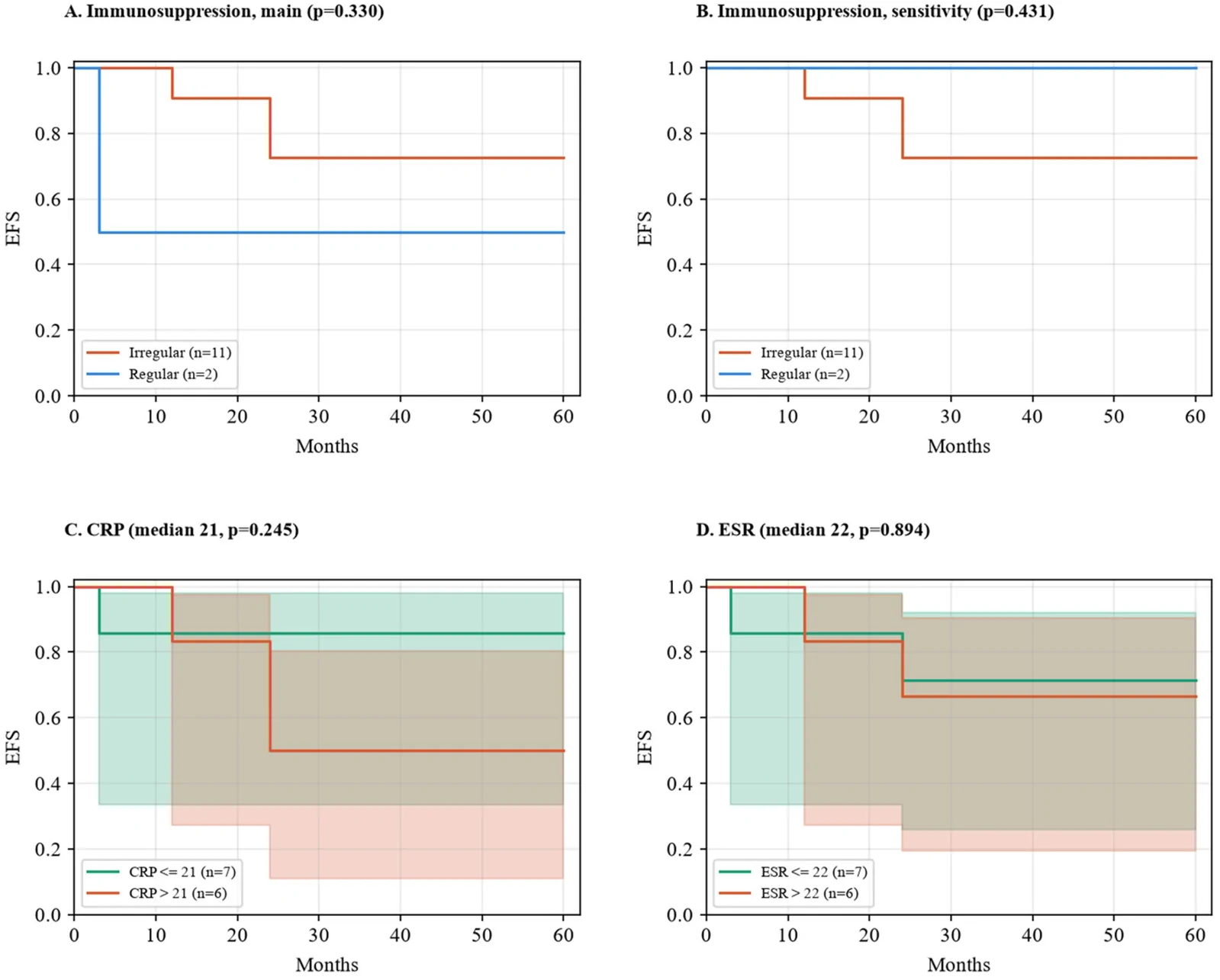
Subgroup analyses of EFS. **(A)** Primary analysis of immunosuppressive adherence: adherent group (*n* = 2) had a 5-year EFS of 50.0% vs. 72.7% in the non-adherent group (*n* = 11), *p* = 0.462. **(B)** Sensitivity analysis of immunosuppressive adherence (excluding technical complications): 5-year EFS in the adherent group rose to 100% vs. 72.7% in the non-adherent group, *p* = 0.431. **(C)** CRP (median 21 mg/L): high group 5-year EFS 50.0% vs. low group 85.7%, *p* = 0.200. **(D)** ESR (median 22 mm/h): high group 66.7% vs. low group 71.4%, *p* = 0.994, no difference.

In the supplementary four-level adherence analysis, no patient met criteria for full perioperative adherence (group A, *n* = 0). In the sensitivity (disease-specific) analysis, 5-year EFS was 100% in group B (preoperative-only, *n* = 2), 50.0% in group C (postoperative-only, *n* = 2), and 77.8% in group D (neither, *n* = 9) (log-rank *p* = 0.355); in the primary analysis the corresponding values were 50.0%, 50.0% and 77.8% (log-rank *p* = 0.602) (Supplementary Figure S1). The numerically highest EFS in group B is directionally consistent with a protective effect of preoperative disease control; however, the extremely small cell counts (2/2/9, with one empty group) preclude any formal inference and these results are descriptive only.

Patients with CRP above the median (21 mg/L) had a 5-year EFS of 50.0% vs. 85.7% for those below (*p* = 0.200); for ESR (median 22 mm/h), the high-value group had 66.7% vs. 71.4% in the low-value group (*p* = 0.994) (Figures 3C,D). For the five derived inflammatory indices (Figures 4, 5): for NLR (median 2.80), PLR (median 98.01), and IBI (median 13.61), the high-value groups each had a 5-year EFS of 50.0% vs. 85.7% in the low-value groups (all *p* = 0.200); for SIRI (median 2.46), the high-value group had 50.0% vs. 85.7% (*p* = 0.131); for SII (median 547.56), the high-value group had 66.7% vs. 71.4% (*p* = 0.787).

**Figure 4:**
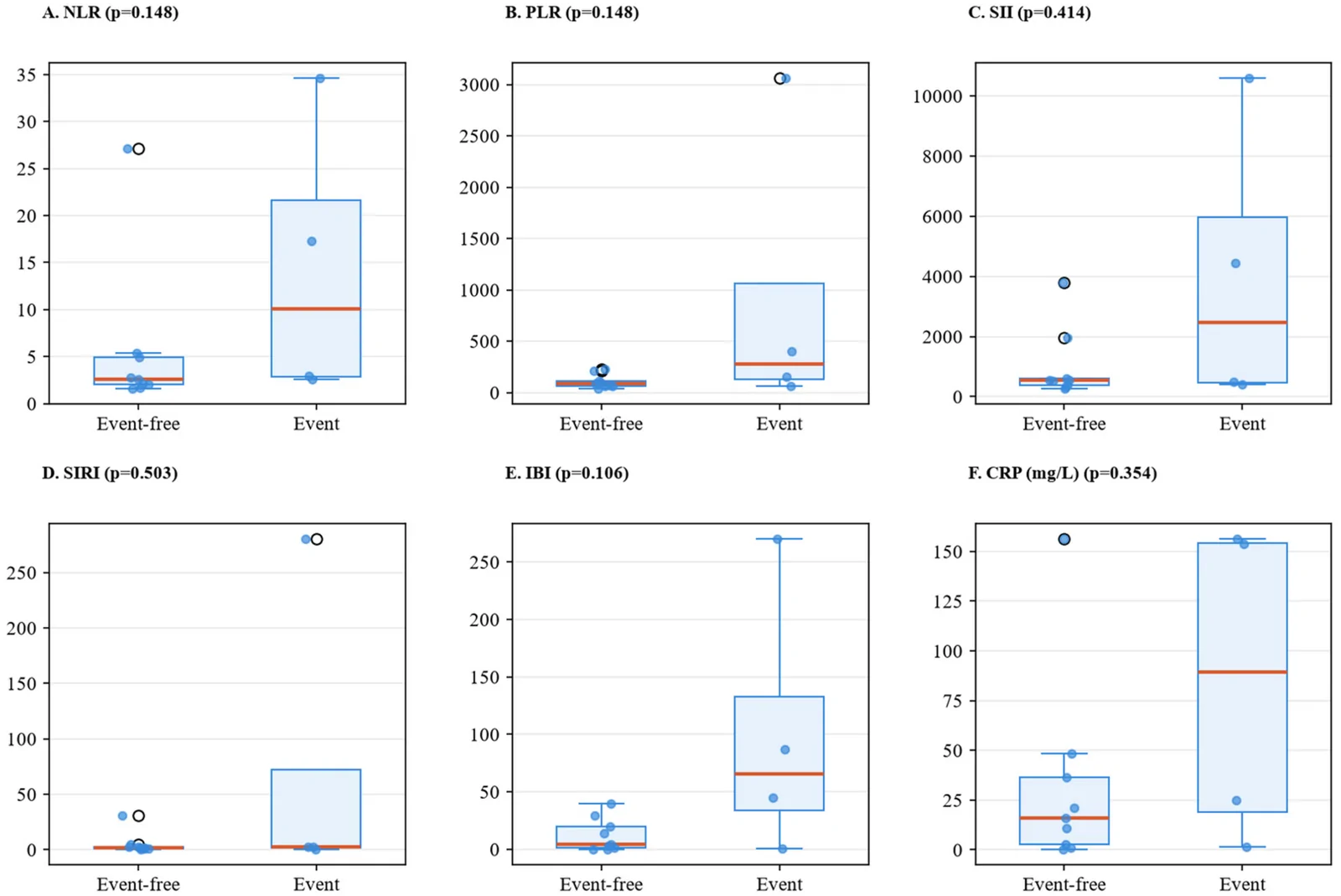
Box plots of derived inflammatory indices (event group vs. event-free group). **(A)** NLR; **(B)** PLR; **(C)** SII; **(D)** SIRI; **(E)** IBI; **(F)** CRP. Boxes represent the interquartile range, central lines indicate medians, and dots represent individual values. *P*-values from the Mann–Whitney *U*-test are shown in each panel.

**Figure 5:**
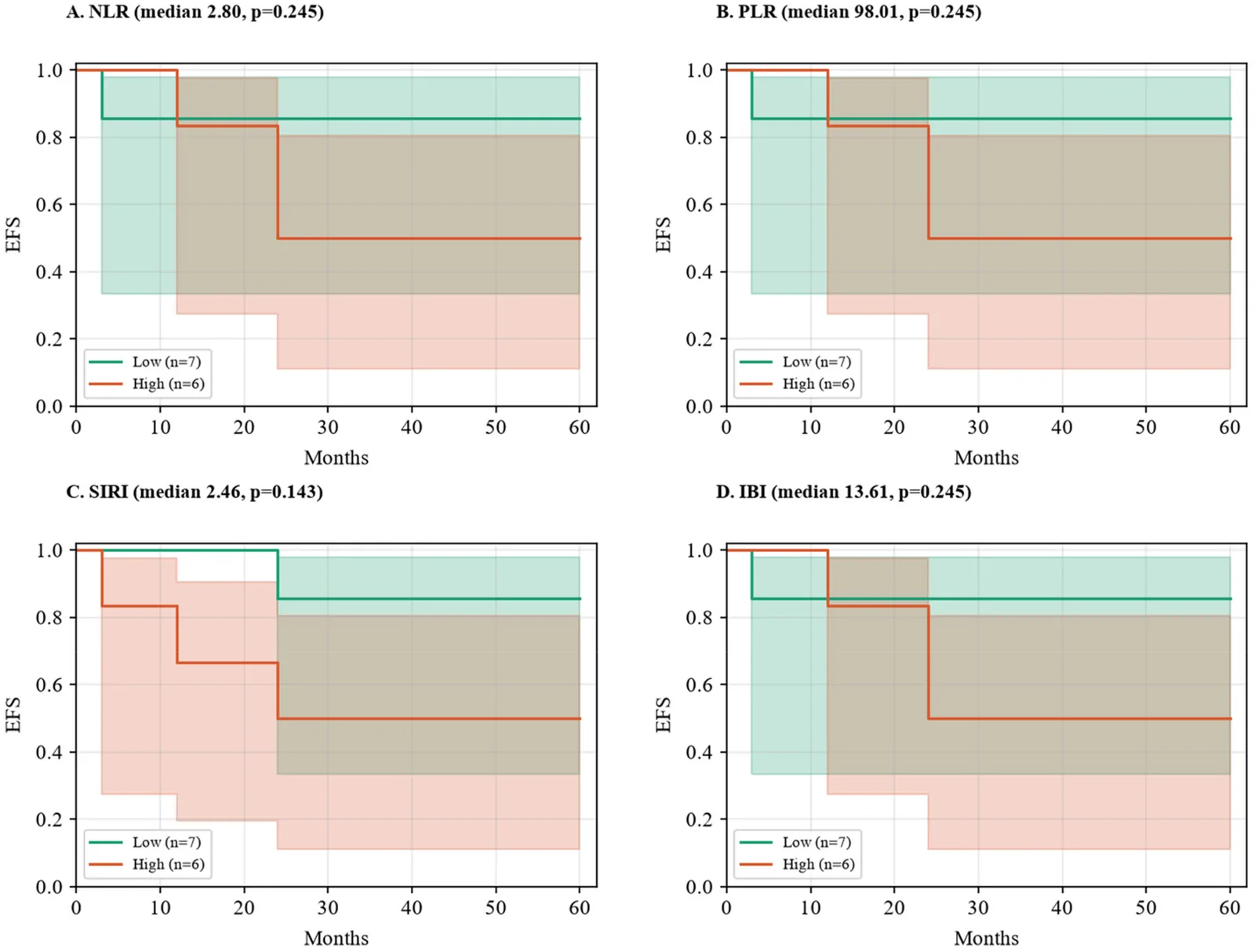
Subgroup EFS by derived inflammatory indices (median) **(A)** NLR (median 2.80, *p* = 0.200); **(B)** PLR (median 98.01, *p* = 0.200); **(C)** SIRI (median 2.46, *p* = 0.131); **(D)** ibI (median 13.61, *p* = 0.200). The high-value groups all had a 5-year EFS of 50.0% vs. 85.7% in the low-value groups, with SIRI showing the most pronounced trend.

Based on the sensitivity analysis (excluding technical complications), three disease-specific events occurred within 5 years (case 2: *in situ* recurrence at 24 months; case 6: *in situ* recurrence at 24 months; case 13: *de novo* occurrence at a new site at 12 months), all in patients with preoperative non-adherence. Case 2 was counted in the primary analysis by the first-event principle (technical complication at 1 month); after exclusion in the sensitivity analysis, the 24-month *in situ* recurrence was re-included. Case 10 was counted in the primary analysis by the technical complication at 3 months; after exclusion, no disease-specific event occurred within 5 years. Three patients developed late *de novo* aneurysms beyond 5 years: case 3 developed a new left superficial femoral artery aneurysm (8.8 × 7.1 cm, treated with intervention) 2 years after drug discontinuation; case 4 developed multiple *de novo* aneurysms (right subclavian + right lower extremity) after drug discontinuation, subsequently resumed immunosuppression; case 10 developed a *de novo* aneurysm after self-discontinuation. The patient with sustained adherence (case 7) remained recurrence-free throughout.

### 3.5 External validation against the global literature (IPD meta-analysis)

A total of 27 published individual cases were pooled with the 13 cases from our cohort (40 cases overall), along with 12 series-level summaries (9, 10, 14–16). In the pooled individual data set, the median age was 35 years, with 82% male; procedure distribution was 12 endovascular, 21 open, and 2 hybrid; there was 1 death among 27 cases (2%) and 0 amputations among 25 cases (0%). A temporal trend was observed: early open series (1989–2011) reported mortality rates of 26%–40% and amputation rates of 22% (7, 17, 18), whereas contemporary endovascular series (2019–2023) reported mortality rates of 0%–5.8% and technical success rates of 69%–100% (19–21) (Figures 6A,B). The distribution of procedure types evolved over time from open dominance to increasing endovascular adoption (Figure 6C).

**Figure 6:**
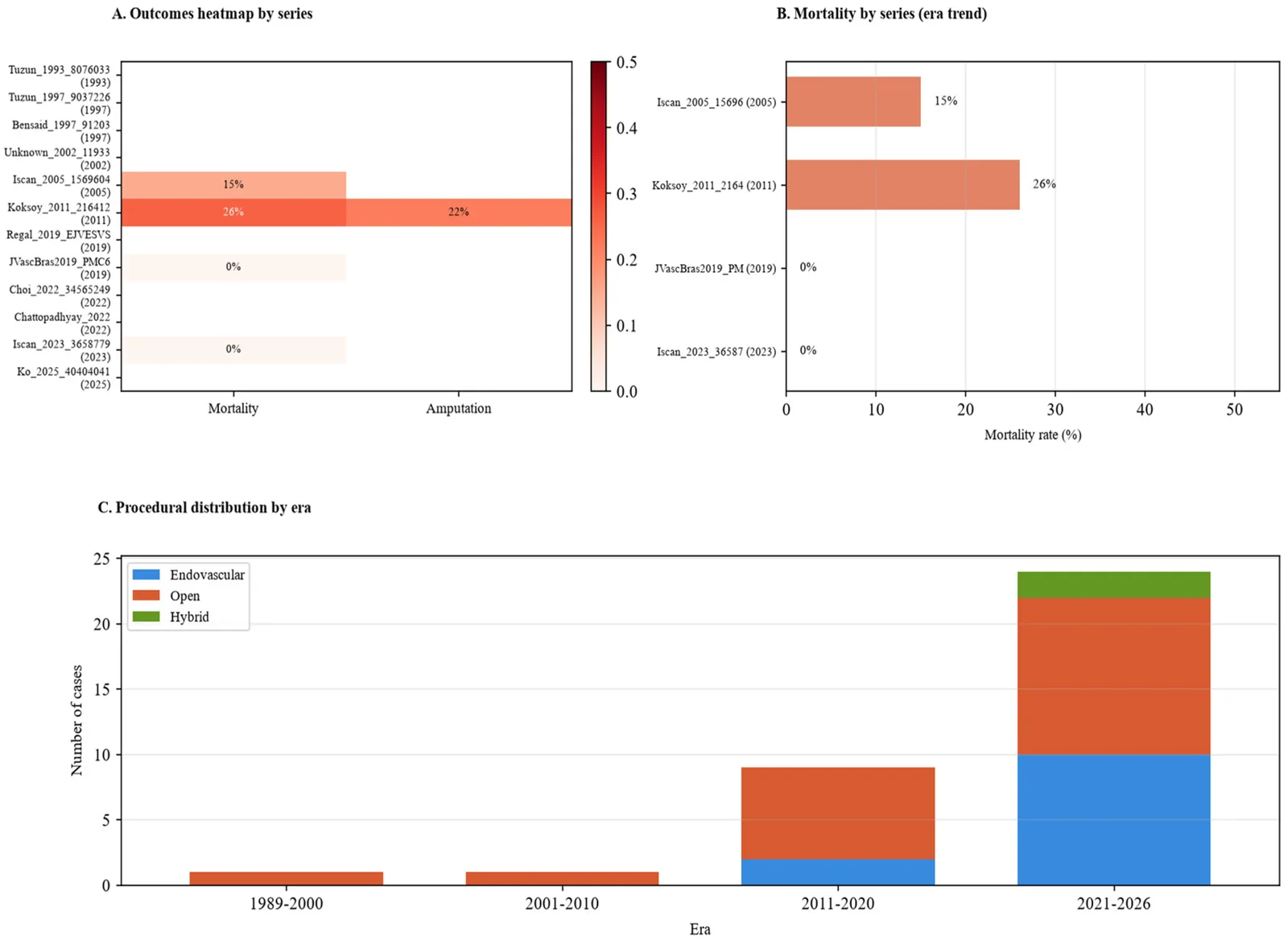
Integrated IPD meta-analysis plots. **(A)** Heatmap of outcomes across case series: rows are ordered by publication year, columns display mortality and amputation rates, with color intensity reflecting the rate values. **(B)** Forest plot (bars) of mortality rates across series, demonstrating the temporal decline from 26%–40% in early open series (1989–2011) to 0%–5.8% in contemporary endovascular series (2019–2023). **(C)** Procedure type distribution by era (stacked bar chart), showing the evolution from open dominance to increasing endovascular adoption over time.

Published individual cases had a median follow-up of only 6 months with a recurrence rate of 11%, whereas our cohort had a median follow-up of 60 months with a recurrence rate of 31% (Table 2).

**Table 2:** Comparison of outcomes between the present cohort and the literature, with summary of inflammatory index subgroups.

Metric	Present cohort (*n* = 13)	Published individual cases (*n* = 27)	Remarks
Median follow-up (months)	60 (36–60)	6 (3–12)	Extended in present cohort
Recurrence/events	4/13 (30.8)	1/9 (11.1)	Underestimated in literature
Late *de novo* (>5 years)	3 (23.1)	—	All occurred with drug discontinuation
Mortality	0/13 (0)	1/27 (3.7)	—
Amputation	0/13 (0)	0/25 (0)	—
5-year EFS (primary/sensitivity)	69.2%/76.9%	—	Short follow-up in literature
Endovascular vs. open (p)	0.992	—	—
CRP high vs. low (p)	0.200	—	Exploratory trend
ESR high vs. low (p)	0.994	—	No difference
SIRI high vs. low (p)	0.131	—	—
NLR/PLR/IBI high vs. low (p)	0.200	—	—
SII high vs. low (p)	0.787	—	—

Data are presented as median (range or IQR), *n*/*N* (%), or *p*-values. EFS, event-free survival; NA, not estimable (due to short follow-up in the literature). Inflammatory index subgroup analyses were based on median grouping using the log-rank test.

## 4 Discussion

This single-center extended follow-up cohort study, integrated with an IPD meta-analysis of 13 BS–FAP patients and 40 published individual cases, yielded four main findings. First, extended follow-up at a median of 60 months revealed a substantially higher recurrence burden than reported in the literature (5-year EFS 69.2%, event rate 31%), whereas published individual cases had a median follow-up of only 6 months and a recurrence rate of 11%, suggesting that the true post-operative recurrence risk of BS–FAP has been systematically underestimated. Second, non-adherence to immunosuppressive therapy was closely associated with late *de novo* aneurysms, and patients with sustained adherence throughout the course remained event-free, positioning adherence as a modifiable target. Third, SIRI showed the most favorable risk-stratification trend (high vs. low: 5-year EFS 50.0% vs. 85.7%, *p* = 0.131), outperforming CRP (*p* = 0.200) and ESR (*p* = 0.994), whereas SII showed no trend (*p* = 0.787). Fourth, endovascular and open repair yielded comparable 5-year EFS (66.7% vs. 66.7%, *p* = 0.992), albeit confounded by selection bias.

The marked discrepancy between short-term and long-term event rates represents the most clinically translatable finding of this study. Published individual cases had a median follow-up of only 3–12 months with a recurrence rate of approximately 11% (5, 6), whereas our cohort had a median follow-up of 60 months with an event rate of 31%. This disparity arises from the chronic, year-scale evolution of BS vascular inflammation (3, 4, 11): in our cohort, the earliest same-site recurrence occurred at 24 months postoperatively, and *de novo* events could occur as late as beyond 5 years, corroborating the concept of a “pan-arterial bed risk” in vasculo-BS (5, 7). The clinical implication is clear: postoperative surveillance should not be limited to 12 months but should be extended beyond 5 years, with vascular imaging (bilateral lower-extremity and aortic ultrasound/CTA) combined with inflammatory marker monitoring every 6–12 months, alongside a proactive follow-up system.

The strong association between non-adherence to immunosuppressive therapy and late recurrence warrants particular attention. All three patients who developed *de novo* aneurysms beyond 5 years postoperatively did so after immunosuppressive discontinuation (cases 3, 4, and 10), and the only patient with sustained adherence (case 7) remained event-free throughout. Case 10, who was adherent preoperatively but discontinued postoperatively, experienced both an early complication and a late *de novo* event, suggesting that preoperative use cannot compensate for postoperative interruption. The underlying mechanism is clear: BS vascular wall inflammation is mediated by T cells, neutrophils, and monocytes, and glucocorticoids combined with DMARDs effectively suppress the inflammatory microenvironment (3, 4, 11) upon interruption, inflammation rebounds rapidly, and vessel wall remodeling often remains asymptomatic until pseudoaneurysm formation ensues. This finding elevates adherence to a therapeutic dimension on a par with surgical repair, mandating the establishment of a joint vascular surgery–rheumatology follow-up pathway and the incorporation of adherence into core postoperative quality metrics.

Although SIRI did not reach statistical significance (*p* = 0.131), it emerged as the most promising candidate biomarker. SIRI integrates neutrophils, monocytes, and lymphocytes, capturing a more comprehensive inflammation–immune imbalance than any single marker: neutrophils mediate acute inflammation, monocytes reflect chronic inflammation and macrophage differentiation, and lymphocytes represent adaptive immune dysregulation (Th1/Th17 predominance being a hallmark of BS immunopathology) (3, 4). The ratio of these three lineages may detect subclinical inflammation even when CRP is normal. This biological plausibility is consistent with reports in other diseases showing that SIRI outperforms NLR and SII (12, 13, 22). By contrast, SII showed no trend (*p* = 0.787), possibly because platelets are subject to multiple modulatory influences (anticoagulation, D-dimer, and disease activity), and noise may have masked the true association in a small sample. CRP showed a consistent directional trend (*p* = 0.200), whereas ESR had no discriminatory capacity (*p* = 0.994). SIRI, derived from routine blood counts at no additional cost, is suggested as an adjunctive postoperative risk-stratification tool, although external validation is required. For patients with SIRI > 2.46, shortening imaging intervals to 6 months and intensifying immunosuppressive assessment may be considered.

Endovascular and open repair yielded comparable 5-year EFS (*p* = 0.992), consistent with Ko et al. (2), and the IPD meta-analysis also demonstrated a temporal trend (early open mortality 26%–40% vs. contemporary endovascular 0%–5.8%) (7, 17–21). However, this equivalence should be interpreted with caution: in our cohort, the endovascular group was younger (32 vs. 43 years) and had a lower inflammatory burden (CRP 6.1 vs. 36.8 mg/L, SIRI 1.85 vs. 2.80), representing a clear selection bias. Against this background, “no difference” may even suggest that open repair has advantages in managing complex inflammation, or it may simply reflect the inability of a small sample to detect a true difference. In practice, the core determinant of procedure selection should be inflammation control to a safe level, rather than technical preference.

The IPD meta-analysis provided important external context. Pooling 27 published individual cases and 12 case series clearly illustrated the temporal evolution (9, 10, 14–16): early open series reported mortality of 26%–40% and amputation of 22% (7, 17, 18), whereas contemporary endovascular series reported mortality of 0%–5.8% and technical success of 69%–100% (19–21). This improvement reflects advances in endovascular technology and standardization of perioperative immunosuppressive management. The stark contrast between a median follow-up of only 6 months in published cases and 60 months in our cohort further confirms that the true recurrence burden has been systematically underestimated. This finding suggests that rare disease reports should specify a minimum follow-up duration and clearly document immunosuppressive regimens and adherence.

Generalizability and next steps. We acknowledge that the small, single-center cohort and the selection biases discussed above limit the generalizability of our findings. The effect-size estimates from this exploratory study may serve as planning parameters for future multicenter prospective studies. External validation of SIRI (and of NLR, PLR and IBI) as postoperative risk-stratification biomarkers will require a prospective multicenter registry with harmonized blood-count timing, standardized imaging surveillance, and adequate events to support multivariable adjustment. Standardization of immunosuppressive regimens is another priority: the agents used in this cohort were heterogeneous, and prospective protocols should align with the EULAR recommendations (4), evaluating whether TNF-inhibitor-based regimens confer lower recurrence than colchicine or conventional DMARDs. Regarding postoperative surveillance, we propose a structured protocol: bilateral lower-limb and aortic imaging (ultrasound and/or CTA) combined with inflammatory-marker monitoring every 6 months for the first 2 years and every 6–12 months thereafter, extending beyond 5 years, integrated within a joint vascular-surgery-rheumatology clinic-in keeping with the reviewer's recommendation for rheumatology collaboration in analyzing disease phenotypes and immunosuppressive management.

Several limitations should be acknowledged. First, the sample size is small (*n* = 13), an inherent constraint of this double-rare entity, with only 4 events precluding multivariate regression; all subgroup comparisons were unadjusted univariate analyses, and this study is explicitly positioned as exploratory and hypothesis-generating. The estimated effect sizes (SIRI risk difference of approximately 35 percentage points and non-adherence risk difference of approximately 27 percentage points) may serve as parameters for sample-size calculations in future multicenter studies; we agree with the reviewer that a multicenter study is ultimately needed and have proposed an international collaborative registry (see Conclusion). Second, the single-center retrospective design introduces selection and referral bias. Third, only patients who underwent surgical treatment were included; patients managed conservatively or with percutaneous drainage alone were excluded by design, and as immunosuppressive therapy is a cornerstone of vascular BS management, our estimates reflect the surgically treated subset and may not generalize to the full spectrum of BS-FAP. Fourth, five patients were excluded owing to missing baseline complete blood count or CRP values precluding calculation of the derived indices; although this exclusion was determined by data availability rather than clinical outcome and the direction of any resulting bias is unlikely to alter the exploratory conclusions, a degree of information bias cannot be fully excluded. Fifth, the IPD meta-analysis was limited by missing immunological details and short follow-up in the literature. Sixth, inflammatory indices were measured only once preoperatively, without assessment of postoperative dynamic changes; a single snapshot measurement may be affected by non-disease-related factors (infections, medications, nutrition) and its ability to predict long-term outcomes requires prospective validation with serial measurements. Seventh, the specific immunosuppressive agents, doses and durations were not systematically recorded; we therefore cannot directly compare TNF-inhibitor-based regimens against colchicine or conventional DMARDs, which may differ in preventing relapse. Eighth, postoperative anticoagulation (agent, duration, and whether administered to all patients or only to those with stents) was not collected systematically and could not be analyzed in relation to in-stent or graft thrombosis. Ninth, the dichotomous adherence classification did not capture gradients; the supplementary four-level analysis remained constrained by empty/small cells. Tenth, lymphocyte counts were approximated, which may slightly overestimate values, although the direction of bias would be consistent. Despite these limitations, the extended follow-up, consistent directional trends across multiple analyses, and biological plausibility collectively support that this signal warrants validation in larger samples.

In conclusion, BS–FAP carries a substantial long-term recurrence burden that is systematically underestimated by short-term follow-up. Immunosuppressive adherence is a modifiable target, SIRI is a promising candidate biomarker requiring external validation, and endovascular and open repair yield comparable long-term EFS, although interpretation should account for selection bias. These findings support establishing a comprehensive management pathway of “surgical repair—uninterrupted immunosuppressive maintenance—extended surveillance ≥5 years,” with adherence incorporated as a core quality metric. The development of a multicenter international collaborative registry with standardized collection of surgical details, immunosuppressive regimens, inflammatory indices, and harmonized follow-up data is an essential next step to validate these findings and generate higher-level evidence.

## Data Availability

The original contributions presented in the study are included in the article/Supplementary Material, further inquiries can be directed to the corresponding author.
